# Combined Tricuspid Valve Repair and Orthotopic Liver Transplantation in a Patient With Severe Tricuspid Regurgitation and Pulmonary Hypertension

**DOI:** 10.7759/cureus.28146

**Published:** 2022-08-18

**Authors:** Nimit Kasliwal, Cheng Yang, Eric J Martinez, Robert F Hebeler, Saravanan Ramamoorthy

**Affiliations:** 1 Anesthesiology, Texas A&M College of Medicine, Dallas, USA; 2 Anesthesiology, Brooke Army Medical Center, San Antonio, USA; 3 Surgery, Baylor University Medical Center, Dallas, USA; 4 Anesthesiology, Baylor University Medical Center, Dallas, USA; 5 Anesthesiology, U.S. (United States) Anesthesia Partners, Dallas, USA

**Keywords:** third degree atrioventricular block, end-stage renal disease, cirrhosis, orthotopic liver transplantation, tricuspid valve repair, tricuspid valve regurgitation

## Abstract

Severe pulmonary hypertension and severe tricuspid regurgitation are often considered strict contraindications for orthotopic liver transplantation. A combined approach of tricuspid repair and subsequent liver transplantation could provide a novel approach for patients with severe pulmonary hypertension and tricuspid regurgitation to undergo orthotopic liver transplantation. A 62-year-old male with a history of end-stage renal disease on hemodialysis, cirrhosis, and third-degree atrioventricular heart block status post single lead pacemaker insertion presented for an orthotopic liver transplant. However, after placement of a Swan-Ganz catheter by the anesthesia team, the patient's central venous pressure was found to be high, and his mean pulmonary artery pressure was 40 mmHg. His case was canceled due to concern for poor postoperative outcomes after a subsequent transesophageal echocardiogram revealed a severely dilated right heart and 4+ tricuspid regurgitation with flow reversal into the hepatic veins. After discussion among the hospital's transplant committee, the patient was planned to have a tricuspid valve repair, liver transplant, and kidney transplant surgery several months later. The patient successfully underwent tricuspid valve repair and orthotopic liver transplant and then kidney transplant the following day.

## Introduction

Traditionally, severe tricuspid regurgitation combined with pulmonary hypertension is associated with increased mortality post-liver transplant. There is a complex interplay between liver disease and pulmonary hypertension that presents a challenge in the prognostication of liver recipient outcomes [1-3]. Severe cirrhosis and advanced liver disease can cause pulmonary hypertension in the case of porto-pulmonary hypertension, and hyperdynamic changes from chronic liver disease can lead to debilitating hypoxemia in the case of hepatopulmonary syndrome. Perplexingly, increasing severity of tricuspid regurgitation is associated with worse post-transplant mortality, and increasing liver disease severity is also associated with worse tricuspid valve repair outcomes [4,5]. This case shows that these two high-risk surgeries can be performed sequentially to mitigate their respective risks against each other.

## Case presentation

**Figure 1:**
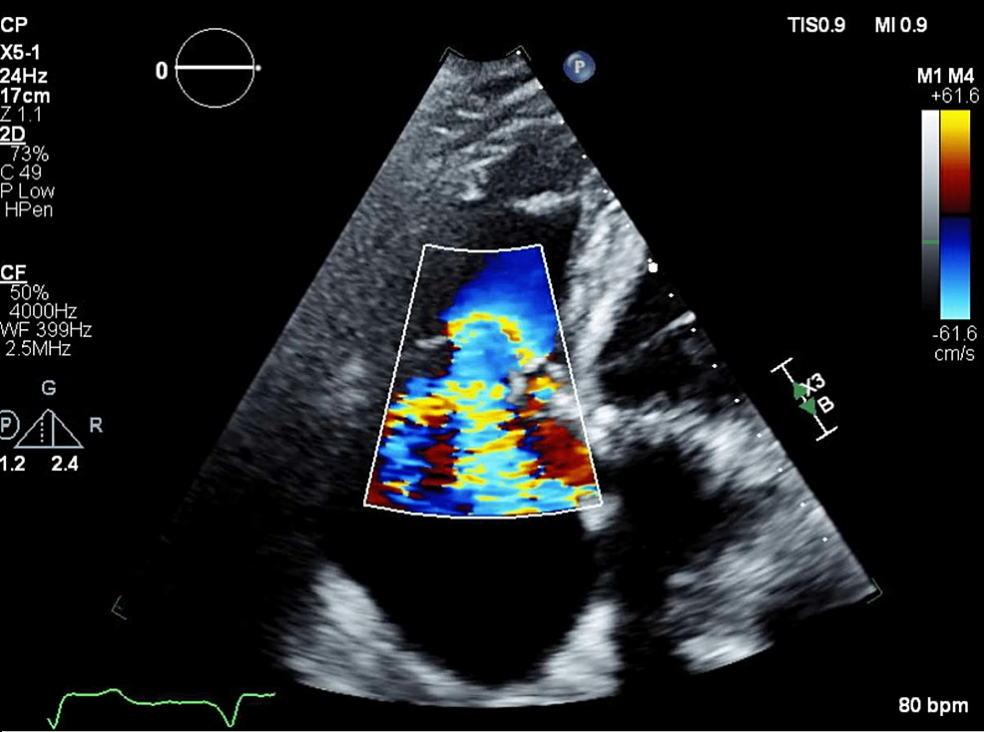
Ultrasonogram showing severe tricuspid regurgitation

A 62-year-old male with a history of end-stage liver disease, end-stage renal disease on scheduled hemodialysis, third-degree atrioventricular block, and idiopathic cardiomyopathy presented for combined tricuspid valve repair, orthotopic liver transplant, and kidney transplant. The patient had presented for an orthotopic liver transplant several months earlier when an intraoperative transesophageal echocardiogram revealed right-sided dilated cardiomyopathy and 4+ tricuspid regurgitation with flow reversal into the hepatic veins. Swan-Ganz catheterization showed a mean pulmonary arterial pressure of 40 mmHg. Following discussion between the anesthesia team and surgical team, his original case was aborted due to concern for poor postoperative outcomes given the new finding of severe tricuspid regurgitation, shown in Figure 1.

Several months later, the patient underwent tricuspid valve repair with a #33 Mosaic porcine xenograft (Medtronic, Minneapolis, United States). The patient was brought to the operating room, general anesthesia was induced, he was endotracheally intubated uneventfully, a central line was placed in the left internal jugular vein, an arterial line was placed in the right brachial artery, and he received intravenous antibiotics. The patient required total cardiopulmonary bypass during the repair of the tricuspid valve. TEE showed no tricuspid regurgitation after repair of the valve and he was weaned easily from bypass with low-dose inotropic agents including milrinone. The patient lost about 300 ml of blood and there was no need for blood products. 

After the tricuspid valve repair was completed and he was deemed stable he underwent an orthotopic liver transplant in the same operating room. During the timeout, the patient's blood type was cross-referenced with the donor blood type to ensure that both were O+. The patient was placed on venovenous bypass prior to performing the hepatectomy. This surgery involved the use of a whole donor liver. Once the donor liver was brought into the field, the surgical team began to anastomose the upper and lower vena cava. Then, the hepatic artery, portal vein, and biliary anastomoses were performed in order. The patient's estimated blood loss was 4.8 L, and thus he required 3 L crystalloid, 2 L colloid, three units packed red blood cells (PRBCs), 10 units fresh frozen plasma (FFP), 40 units cryoprecipitate, two units platelets, and 1200 ml of cell saver. Hemostasis was achieved and the patient tolerated the procedure well and was taken to the ICU in critical, but stable condition on pressor support (epinephrine, vasopressin, norepinephrine) and mechanical ventilation. Graft function was satisfactory with no evidence of rejection or infection. Doppler ultrasound evaluation showed normal post-transplant pathology and was unremarkable. 

On postoperative day one, the patient returned to the operating room for a kidney transplant. As before, during the timeout, the patient's blood type was cross-referenced with the donor blood type to ensure that both were O+. After a nephrectomy was performed, the donor kidney was brought into the field. Anastomoses were performed for the renal vessels, external iliac artery, ureters, and ureteral vessels. A ureteral stent was placed. The patient lost about 200 ml of blood and there was no need for blood products. The patient tolerated the procedure well and was taken to the ICU in stable condition. The patient was sedated with propofol, mechanically ventilated, on continuous renal replacement therapy, and on pressor support (epinephrine, vasopressin, norepinephrine).

After extubation, the patient continued to have worsening altered mental status and delirium with confusion post-surgery while in the ICU. His urine output was slow to pick up initially and he remained on continuous venovenous hemodialysis until his urine output slowly increased. His mental status slowly recovered and he was transferred to the floor for continued recovery. He had difficulty with ileus postoperatively. To rule out a bowel obstruction, an abdominal CT scan was obtained, which revealed normal postoperative ileus. To facilitate proper recovery of bowel function, he was NPO (nothing by mouth), then was slowly started on tube feeds, and soon advanced to a regular diet. His allograft function continued to improve daily. His immunosuppression regimen included tacrolimus 4 mg *bis in die* or two times a day (BID) and mycophenolate 720 mg BID. After 21 days, he continued to be physically debilitated and deconditioned which required transfer to an inpatient rehab facility for two weeks before being discharged home with a plan for home health physical therapy.

His past medical history included end-stage liver disease (Model for End-stage Liver Disease (MELD) score 32), cirrhosis with ascites necessitating frequent paracenteses, end-stage renal disease on scheduled hemodialysis, severe tricuspid regurgitation (flail septal/posterior leaflets, with torn chordae), permanent atrial fibrillation status post ablation, third-degree atrioventricular block status post leadless pacemaker (previously with a single lead pacemaker), idiopathic cardiomyopathy, obstructive sleep apnea, and pulmonary hypertension. Past surgical history included atrial fibrillation ablation, single lead pacemaker placement status post removal due to tricuspid valve incompetence, left arm arteriovenous fistula creation, cervical spine fusion, penile prosthesis, and total knee arthroplasty. Patients medications were amlodipine 10 mg daily, carvedilol 25 mg twice a day, losartan 100 mg daily, warfarin 2.5 mg daily, Levemir® 15 units daily, cinacalcet 30 mg daily, citalopram 40 mg daily, duloxetine 20 mg daily, solifenacin 5 mg daily, tamsulosin 0.4 mg daily, and topiramate 50 mg daily. He had no known drug allergies. He could independently perform activities of daily living. He denied tobacco use, alcohol use, and illicit drug use. On review of systems, he was positive for fatigue, dyspnea, and cough, and was classified as New York Heart Association (NYHA) Class III. 

## Discussion

It is important to determine if patients have any porto-pulmonary hypertension and/or hepatopulmonary syndrome prior to transplant. Although this patient had neither, his acute worsening of tricuspid regurgitation and pulmonary hypertension led to the decision to postpone his case, despite being a good candidate prior. High mean pulmonary arterial pressures are associated with poor outcomes in the liver transplant population [6-8]. For this reason, many patients with severe tricuspid regurgitation and pulmonary hypertension would not be candidates for orthotopic cadaveric liver transplantation. The decision to repair his heart valve lesion immediately prior to his liver transplant ultimately enabled this patient to receive a new liver. His postoperative course was unremarkable as he required minimal pressors and inotropes and was completely weaned off both by postoperative day two. By postoperative day three, he was extubated. This patient required a tricuspid valve repair and liver transplant to be performed but they were mutually contraindicated for reasons stated above. This case report shows that performing these surgeries in sequence can minimize the risks and concerns that are typically associated with this patient scenario. This could prove to be very beneficial for patients with both tricuspid regurgitation/pulmonary hypertension and liver disease requiring a transplant as these patients would typically be deemed not good candidates for surgery. 

## Conclusions

Traditionally, when severe tricuspid regurgitation and severe pulmonary hypertension are identified prior to an orthotopic liver transplant, this is viewed as a contraindication to the surgery. However, this case shows that the liver transplant can be performed successfully when done subsequently after the repair of the tricuspid valve. The goal of this case report is to highlight the phenomenon that high-risk surgeries like tricuspid valve repair, liver transplantation, and kidney transplantation can be completed in succession in order to alleviate the mutual contraindications associated with these surgeries. Further research and case reports would help contribute to clinicians' understanding of this methodology and of other sequential surgeries that may similarly lead to better patient outcomes.
